# Activity of chemotherapy in mucinous ovarian cancer with a recurrence free interval of more than 6 months: results from the SOCRATES retrospective study

**DOI:** 10.1186/1471-2407-8-252

**Published:** 2008-09-01

**Authors:** Sandro Pignata, Gabriella Ferrandina, Giovanna Scarfone, Paolo Scollo, Franco Odicino, Gennaro Cormio, Dionyssios Katsaros, Antonella Villa, Liliana Mereu, Fabio Ghezzi, Luigi Manzione, Rossella Lauria, Enrico Breda, Desiderio Gueli Alletti, Michela Ballardini, Alessandra Vernaglia Lombardi, Roberto Sorio, Giorgia Mangili, Domenico Priolo, Giovanna Magni, Alessandro Morabito

**Affiliations:** 1 Istituto Nazionale Tumori, Napoli, Italy; 2 Policlinico Agostino Gemelli, Ginecologia Oncologica, Roma, Italy; 3 Ospedale Maggiore Policlinico, Mangiagalli e Regina Elena, Clinica Ostetrico-Ginecologica, Milano, Italy; 4 A.O. S. Cannizzaro, Ginecologia ed Ostetricia, Catania, Italy; 5 A.O. Spedali Civili-Università degli Studi di Brescia, II Ginecologia ed Ostetricia, Brescia, Italy; 6 Azienda Ospedaliera Policlinico, II Ginecologia e Ostetricia, Bari, Italy; 7 Azienda Ospedaliera O.I.R.M.-S. Anna, Ginecologica Oncologica, Università di Torino, Italy; 8 Ospedali Riuniti di Bergamo, U.O. di Ginecologia, Bergamo, Italy; 9 Ospedale Policlinico S. Matteo, Ostetrica e Ginecologica, Pavia, Italy; 10 Università dell'Insubria Clinica Ginecologia e Ostetrica, Varese, Italy; 11 Azienda Ospedaliera S. Carlo, Oncologia Medica, Potenza, Italy; 12 Università Federico II, Oncologia Medica, Napoli, Italy; 13 Ospedale S. Giovanni-Fatebene Fratelli-Isola Tiberina, Oncologia Medica, Roma, Italy; 14 A.O. Vincenzo Cervello, Ostetricia e Ginecologia, Palermo, Italy; 15 Istituto Scientifico Romagnolo per lo Studio e la Cura dei Tumori – IRST, Meldola (FC), Italy; 16 Casa di cura Malzoni, Ginecologia Oncologica, Avellino, Italy; 17 CRO AVIANO, Oncologia Medica, Aviano, Italy; 18 Ospedale S. Raffaele, Ginecologia Oncologica Medica, Milano, Italy; 19 Ospedale S. Vincenzo, Oncologia Medica, Taormina, Italy; 20 QBGROUP spa, Padova, Italy

## Abstract

**Background:**

Mucinous ovarian carcinoma have a poorer prognosis compared with other histological subtypes. The aim of this study was to evaluate, retrospectively, the activity of chemotherapy in patients with platinum sensitive recurrent mucinous ovarian cancer.

**Methods:**

The SOCRATES study retrospectively assessed the pattern of care of a cohort of patients with recurrent platinum-sensitive ovarian cancer observed in the years 2000–2002 in 37 Italian centres. Data were collected between April and September 2005. Patients with recurrent ovarian cancer with > 6 months of platinum free interval were considered eligible.

**Results:**

Twenty patients with mucinous histotype and 388 patients with other histotypes were analyzed. At baseline, mucinous tumours differed from the others for an higher number of patients with lower tumor grading (p = 0.0056) and less advanced FIGO stage (p = 0.025). At time of recurrence, a statistically significant difference was found in performance status (worse in mucinous, p = 0.024). About 20% of patients underwent secondary cytoreduction in both groups, but a lower number of patients were optimally debulked in the mucinous group (p = 0.03). Patients with mucinous cancer received more frequently single agent platinum than platinum based-combination therapy or other non-platinum schedules as second line therapy (p = 0.026), with a response rate lower than in non-mucinous group (36.4% vs 62.6%, respectively, p = 0.04). Median time to progression and overall survival were worse for mucinous ovarian cancer. Finally, mucinous cancer received a lower number of chemotherapy lines (p = 0.0023).

**Conclusion:**

This analysis shows that platinum sensitive mucinous ovarian cancer has a poor response to chemotherapy. Studies dedicated to this histological subgroup are needed.

## Background

Mucinous carcinoma of the ovary accounts for 5–10% of all primary epithelial ovarian cancer [1]. Patients with mucinous ovarian cancer generally undergo the same first- and second-line treatment as patients with other histological subtypes [2]. However, very few reports in the literature have been published on this topic and activity of chemotherapy has been described in a limited number of patients and only in the first-line setting [3-6]. It has recently been shown in two different series of 27 and 45 patients, that advanced mucinous ovarian carcinoma have a poor response to first line chemotherapy [3,6]. Thus, resistance to chemotherapy has been claimed as one of the main cause of the worse prognosis of mucinous ovarian cancer [3].

The SOCRATES (**S**tudy of an **O**varian **C**ancer cohort **R**ecurred **A**fter first-line **T**reament: a r**E**strospectivy **S**urvey) study was planned to retrospectively assess the pattern of care of patients with recurrent platinum-sensitive ovarian cancer observed in Italy in the years 2000–2002 [7]. Using this cohort of patients we evaluated the response of mucinous cancer to chemotherapy in the recurrent setting.

## Methods

Patients with recurrent advanced ovarian cancer and a recurrence free interval (RFI) longer than 6 months were considered eligible for the study. The patients were observed in the years 2000–2002 in 37 Italian centres. Data were collected between April and September 2005. Four-hundred-ninety-three patient files were screened and 408 were considered eligible and analyzed in the present study.

The descriptive analysis of the data has been performed in 2 different subgroups identified according to histology: mucinous cancer and non-mucinous cancer. No central pathology assessment of the cancer samples was done.

Clinical, pathological and treatment characteristics at initial diagnosis, as well as at recurrence, including surgical and medical treatment (up to 6 lines of chemotherapy) of the recurrence were considered. Response rate was calculated considering RECIST [8] or Ca 125 criteria [9].

Overall survival was defined as the time elapsed between recurrence diagnosis and the date of death or the date of last follow-up information for live patients. Time to progression and overall survival were described y the Kaplan-Meier product limit method [10].

Differences among baseline variables were analyzed by the Student t test and Wilcoxon rank test for quantitative variables, and by the Mantel Haenszel test and the Chi-square method for the qualitative variables. Differences were considered statistically significant when *p *< 0.05.

All analysis was done using SAS^® ^(SAS Institute Inc., Cary, NC, USA-version 9.1.3) statistical software.

## Results

Mucinous tumors were diagnosed in 20 patients, as compared with 388 patients with other histological subtypes (table 1). Median age, performance status, results of primary surgery were similar between the two groups. In mucinous ovarian cancer, the grading of the tumors was lower than in the other subtypes (*p *= 0.0056) and stage at diagnosis was less advanced (p = 0.025)

**Table 1:** Characteristics of the patients with recurrent mucinous ovarian cancer compared to other histological subtypes at the time of initial diagnosis of ovarian cancer.

	**Mucinous**	**Other histotypes**	**p**
**Number of patients**	20	388	

**Age (years)**	20	384	n.s.*
Mean (± s.d.)	54.9 ± 12.5	57.7 ± 10.8	
median	55	57	
range	25–71	31–94	

**FIGO stage at diagnosis**	20	383	
I	20.0%	6.5%	0.0258**
II	10.0%	7.3%	
III	65.0%	77.0%	
IV	5.0%	9.1%	

**Grading**	12	348	
1	25.0%	3.4%	0.0056**
2	16.7%	24.7%	
3	58.3%	71.8%	

**Result of cytoreductive surgery**	15	319	
No residual disease	46.7%	26.0%	n.s.**
Optimal (≤ 1 cm residual disease)	20.0%	29.5%	
Suboptimal (> 1 cm residual disease)	33.3%	44.5%	

**ECOG performance status**	15	347	
0	60.0%	68.3%	n.s.**
1	33.3%	26.5%	
2	6.7%	4.3%	
3	.	0.9%	

**Type of first line chemotherapy**	20	338	n.s.***
Platinum single agent	15.0%	11.1%	
Platinum based combinations	85.0%	88.9%	

• t-test; ** χ^2 ^Mantel-Haenszel; *** χ^2^

The main characteristics of the patients at time of recurrence are shown in table 2. A statistically significant difference was found in performance status, that was worse in the mucinous group (p = 0.024), while no differences were found in the number of disease sites, age and recurrence free interval.

**Table 2:** Characteristics of patients with mucinous ovarian cancer with a recurrence free interval > 6 months compared to other histological subtypes at the time of the diagnosis of recurrence.

	**Mucinous**	**Other histotypes**	**p**
**Number of patients**	20	388	

**Age (years)**	20	380	
Mean (± s.d.)	57.6 ± 12.8	59.8 ± 10.8	n.s.*
Median	58	60.5	
Range	28–78	33–97	

**PS Ecog**	20	360	
0	45.0%	62.8%	0.0241**
1	40.0%	33.9%	
2	15.0%	3.3%	
3	.	.	.

**Recurrence free interval **(N)	17	378	
6–12 months	58.8%	38.9%	n.s.**
> 12 months	41.2%	61.1%	
**median (range) **– *months***:**	10.6 (5–141)	15.3 (5–160)	n.s.****

**Number of disease sites**	20	335	
1	25.0%	44.8%	n.s.**
> 1	75.0%	55.2%	

**Surgery**	19	375	
Yes	26.3%	20.5%	n.s.***
No	73.7%	79.5%	

**Result of cytoreductive surgery**	5	64	
No residual	.	50.0%	0.0308***
Residual desease	100.0%	50.0%	

**Ca 125**	14	247	n.s.**
Normal: ≤ 35	14.3%	12.1%	
> 35 U/ml	85.7%	87.9%	

* t-test; ** χ^2 ^Mantel-Haenszel; *** χ^2^; ****Wilcoxon

About 20% of patients underwent secondary cytoreduction in both groups, with a lower number of patients optimally debulked (no residual disease) in the group of patients with mucinous cancer (p = 0.03). The majority of patients with mucinous tumours had increased CA 125 levels at recurrence (85%).

**Figure 1:**
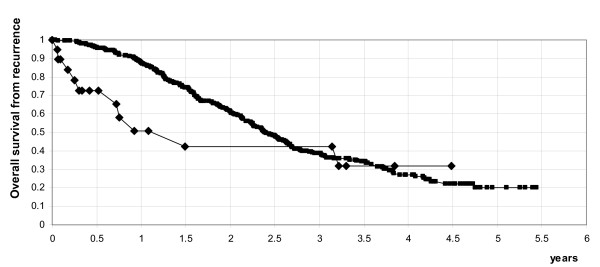
Overall survival from recurrence in patients with mucinous (◆) compared to other histotypes (■).

Details on second-line chemotherapy are shown in the table 3. Patients with mucinous cancer received as second line therapy more frequently single agent platinum (42.1%) than platinum-combination therapy (31.6%) or other non-platinum chemotherapy (26.3%) (p = 0.026). The response rate (CR + PR) to the second line chemotherapy was lower in mucinous cancer than in non-mucinous one (36.4% vs 62.6%, respectively, p = 0.04). Moreover, patients with mucinous cancer received a lower number of lines of chemotherapy as compared to the other histotypes (p = 0.0023). Median progression free survival was 4.5 months in the mucinous and 8 months in non-mucinous group (p = 0.0292). Overall median survival from recurrence was 17.9 months in the mucinous and 28.8 months in non-mucinous group (p = 0.0028) (Figure 1).

**Table 3:** Response to second line chemotherapy in patients with mucinous ovarian cancer with a recurrence free interval > 6 months compared to other histological subtypes

	**Mucinous**	**Other histotypes**	**p**
**N pts.**	20	388	

**Type of second line chemotherapy**	19	384	
Platinum single agent	(8) 42.1%	(67) 17.4%	0.0259*
Platinum based combination	(6) 31.6%	(184) 47.9%	
No platinum	(5) 26.3%	(133) 34.6%	

**Response rate to second line**			
**All evaluable patients**	11	340	
CR + PR	(4) 36.4%	(213) 62.6%	0.0407*

**Platinum**	7	227	
CR + PR	(4) 57.1%	(170) 74.9%	n.s.*

**Non-platinum**	4	113	
CR + PR	(-) 0%	(43) 38.1%	n.s.*

**Number of chemotherapy lines received for recurrence**			
Mean (± s.d.)	1.9 ± 1.1	2.8 + 1.3	0.0023**

* χ^2^; **t-test

In the mucinous cancer group responses were obtained with carboplatin, cisplatin, and carboplatin/paclitaxel (2 responses in patients with 6–12 months and 2 responses in patients with > 12 recurrence free interval). Among patients treated with non platinum-agents, no response was observed at second line, while responses were achieved in third-fourth line with paclitaxel (1/2 patients), topotecan (1/4 patients) and cyclophosphamide (1/1); no activity was observed with liposomal doxorubicin (0/4 patients) and gemcitabine (0/1 patient).

## Discussion

This retrospective study indicates that recurrent mucinous ovarian cancer has a lower response rate to chemotherapy and a worst prognosis compared to non-mucinous subtypes. Moreover, patients receive less chemotherapy lines for recurrence as compared to other histotypes and when undergo secondary cytoreduction, this is less effective. At our knowledge, this analysis describes for the first time the response rate to second line chemotherapy in patients with platinum sensistive mucinous ovarian cancer. At baseline, the only main characteristic differentiating mucinous from non-mucinous tumour was the lower grade of the cancer, according to what previously observed [4]. Although we have not enough data to state that the poor response to chemotherapy is related to the lower grade of the tumours, it is possible to speculate that recurrent low grade cancer may benefit from a more aggressive attempt of cytoreduction before medical therapy. Unfortunately, in our series the patients that underwent secondary cytoreduction did not achieve the goal of obtaining an absence of residual disease; of course, the small number of patients does not allow to reach a definitive conclusion regarding the role of surgery in the treatment of recurrent mucinous ovarian cancer. No other disease related characteristics differed between mucinous and non-mucinous patients at recurrence.

Mucinous carcinomas of the ovary includes 5–10% of ovarian carcinomas, although recent refinements in the interpretation of the histological features of noninvasive and metastatic mucinous carcinomas suggest that this may be an overestimate [1,11]. Clinical stage is the most important predictor of survival in mucinous ovarian carcinoma. The early stages confer a better overall prognosis for survival [11,12], while the advanced disease has been associated with a poorer survival compared to the other histological subgroups [11-13].

The rarity of the disease is the main reason of the paucity of literature data regarding the activity of chemotherapy in this entity. Cloven [14] have shown, *"in vitro"*, that the frequency of extreme drug resistance to chemotherapeutic agents differs significantly among histological subtypes of epithelial ovarian cancer. These authors demonstrated that mucinous ovarian cancer cells are more frequently resistant to cisplatin, but less frequently resistant to topotecan and doxorubicin compared to papillary serous tumors [14], however clinical data are lacking.

In a case-controlled study Hess [3] showed, on 27 mucinous and 54 other histological types,, that patients with advanced mucinous ovarian cancer have a poorer response to platinum-based first-line chemotherapy compared with patients with other histological subtypes, along with a worse survival. In this series, only 37% of the patients were treated with carboplatin/paclitaxel combination as first-line treatment, while the remainder received carboplatin alone or platinum plus anthracyclines. The overall response rate was 26% in first-line chemotherapy, while the response rate in second- and third-line chemotherapy was not reported [3]. A poor response to first line chemotherapy has been described by the Hellenic Cooperative Group [6]. In a previous study, we also showed in 21 consecutive patients with mucinous ovarian cancer treated in a single institution that the response rate to first line chemotherapy was significantly lower than that found in the other histological subgroups, with paclitaxel being the only drug showing activity in second line [4].

Platinum-sensitive recurrent ovarian cancer is usually treated with carboplatin/paclitaxel or carboplatin/gemcitabine, based on the trials showing superiority of combination chemotherapy versus single agent carboplatin [15,16]. In our study an higher proportion of patients with mucinous cancer was treated at recurrence with single agent platinum than with platinum based combination therapy or other non platinum agents. Data clearly indicate that patients with recurrent mucinous ovarian cancer with a recurrence free interval higher than 6 months can respond to a platinum re-treatment, although the response rate is lower than that observed in non-mucinous cancer. Overall, recurrent mucinous cancer patients receive less chemotherapy lines than the others, probably also due to the lack of data in the literature showing activity for the chemotherapy agents more frequently used in this disease.

Here we report for the first time some responses to paclitaxel, topotecan and cyclophosphamide, while no response was observed with liposomal doxorubicin and gemcitabine. Overall the response rate to non-platinum agents was quite poor.

A possible limitation of our report is the retrospective nature of the analysis: therefore, survival data should be interpreted with caution. Another weakness of the study may be the lack of a central pathology review, to confirm these were mucinous ovarian cancers versus metastatic malignancies of gastrointestinal origin. However, the differential diagnosis between gastrointestinal and ovarian cancer is a major problem at time of initial diagnosis. In fact, in the case of our series of recurrent ovarian cancer this limitation may be less important since it is likely that during the disease free interval the potential presence of a primary gastrointestinal cancer would have been diagnosed. Moreover, a worse performance status was found in patients with mucinous tumors: however, due to the small number of patients, no definite conclusions can be drawn regarding the potential effect of performance status on the poor survival of patients with mucinous tumors.

Conventional parameters used to predict the clinical behaviour of advanced ovarian cancer may not adequately correlate with prognosis in mucinous carcinoma. Several studies have shown that mucinous ovarian cancer has a different pattern of expression of some molecular factors compared to the other subtypes. It is possible that a better understanding of tumour biology may help in determining which patients with mucinous ovarian cancer would benefit from traditional chemotherapy or should receive alternative chemotherapy agents. Several studies have shown that RAS mutations (specifically at KRAS codon 12) are prevalent in ovarian cancers of mucinous histology but not in tumors of non-mucinous histologies [17,19]. On the contrary, mutation of p53, which is considered important in defining sensitivity to paclitaxel, is less frequent in mucinous tumors [20]. Again, some studies have found that the expression of COX-2 was much less frequent in mucinous cancer than in serous and endometroid ovarian cancers [21,22]. Chemotherapy decisions tailored to the biology of mucinous ovarian cancer should be investigated in the future. The rarity of the disease should not discourage the assessment, in clinical trials, of the activity of different drugs, choosing first among those active in gastrointestinal cancer. Furthermore, *"in vitro" *drug response assays could be very useful to select patients that are likely to be resistant to traditional chemotherapy for whom to suggest an alternative, experimental treatment.

## Conclusion

In conclusion, we showed that mucinous ovarian cancer has a poor response to chemotherapy in the recurrence setting along with a worst prognosis. Responses to platinum re-treatment are less frequent than in non-mucinous cancer, while anecdotal responses occur with non-platinum agents. Studies with alternative chemotherapy combinations are mandatory in this histological subgroup.

## Competing interests

The authors declare that they have no competing interests.

## Authors' contributions

SP, GF, GS, PS participated in the design of the study; GM performed the statistical analysis. SP conceived of the study, and participated in its design and coordination. FO, GC, DK, AV, LM, FG, LM, RL, EB, DGA, MB, AVL, RS, GM, DP, AM significantly contributed to data collection. All authors read and approved the final manuscript. Additional co-authors and participating institution are listed in the additional file 1.

## Pre-publication history

The pre-publication history for this paper can be accessed here:



## Supplementary Material

Additional file 1 participating institutions and co-authors. Click here for file
