# Supplementary material for: Activity of chemotherapy in mucinous ovarian cancer with a recurrence free interval of more than 6 months: results from the SOCRATES retrospective study
Source: BMC Cancer. 2008 Sep 1;8:252. doi: 10.1186/1471-2407-8-252 (PMC2538544; doi:10.1186/1471-2407-8-252)
Supplement: Additional file 1 — participating institutions and co-authors. [file 1471-2407-8-252-S1.doc]

Participating Institutions and coauthors

| **Hospital** | **Division** |  |  |
| --- | --- | --- | --- |
| A.O. Spedali Civili di Brescia | II ginecologia ed ostetricia | Sergio Pecorelli | Franco Odicino |
| Policlinico Agostino Gemelli  Roma | Ginecologia Oncologica | Giovanni Scambia | Gabriella Ferrandina |
| Policlinico Bari | II° Ginecologia e Ostetricia | Luigi Selvaggi | Gennaro Cormio |
| Istituto Nazionale Tumori, Napoli | Oncologia Medica B,Ginecologia, UOC Sperimentazioni Cliniche | Sandro Pignata Francesco Perrone  Alessandro Morabito | Carmela Pisano  Stefano Greggi  Francescapaola Magazzino |
| Azienda Ospedaliera O.I.R.M.-S. Anna, Università di Torino | Ginecologia Oncologica | Marco Massobbio  Manuela Puopolo | Dionyssios Katsaros |
| Ospedali Riuniti di Bergamo | Ginecologia | Luigi Frigerio | Antonella Villa |
| Ospedale Policlinico S. Matteo, Pavia | Ostetrica e Ginecologica | Emilio Imparato | Saverio Tateo  Liliana Mereu |
| Ospedale F. Del Ponte, Varese | Clinica Ostetrica-Ginecologia | Pierfrancesco Bolis | Fabio Grezzi  Nicoletta Donadello |
| Clinica Mangiagalli, Milano | Clinica Ostetrico-Ginecologica | Giorgio Bolis | Giovanna Scarfone |
| AO S. Carlo, Potenza | Oncologia Medica | Luigi Manzione | Rosangela Romano |
| Policlinico Federico II, Napoli | Oncologia Medica | Sabino De Placido | Rossella Lauria  Emilia Montagna,  Emilia Buccino,  Valeria Forestieri |
| Ospedale S. Giovanni- Fatebene Fratelli - Isola Tiberina, Roma | Oncologia Medica | Vittorina Zagonel | Enrico Breda |
| A.O. Vincenzo Cervello, Palermo | Ostetricia -ginecologia | D Gueli Alletti | Giovanna Marforio |
| Ospedale Morgagni- Pierantoni, Forlì | Unità di Biostatistica – Oncologia Medica | Ruggero Ridolfi | Dino Amadori |
| Casa di cura Malzoni, Avellino | Ginecologia Oncologica | Carmine Malzoni | Alessandra Vernaglia Lombardi |
| CRO Aviano | Oncologia Medica | Andrea Veronesi | Roberto Sorio |
| Azienda Ospedaliera S.Maria degli Angeli, Pordenone | Oncologia Medica | Salvatore Tumolo |  |

| Ospedale San Raffaele, Milano | Ginecologia | Augusto Ferrari | Giorgia Mangili |
| --- | --- | --- | --- |
| Ospedale S. Vincenzo, Taormina | Oncologia Medica | Francesco Ferraù | Domenico Priolo |
| A.O. Civico Benfratelli M. Ascoli e G. di Cristina, Palermo | Ginecologia Oncologica | Franco Romano | Pietro Musso |
| Azienda Ospedaliera, Parma | Ginecologia ed Ostetricia | Mauro Melpignano |  |
| Ospedale Civile, Voghera | Oncologia Ginecologica | Giovanni Aspesi | Mauro Presti |
| A.O. Bianchi-Melacrino-Morelli, Reggio Calabria | Oncologia Medica | Mario Nardi | Antonella Falzea |
| Ospedale Generale S. Cuore di Gesù, Benevento | Oncologia Medica | Antonio Febbraro |  |
| Ospedale Mater Salutis, Legnago | Oncologia Medica | Andrea Bonetti | Lara Furini |
| Presidio Ospedaliero di Bentivoglio | Oncologia Medica | Maria Luisa Geminiani | Vincenzo Arigliano |
| Ospedale Belluria di Bologna | Oncologia Medica | Lucio Crinò | Simonetta Rimondini |
| Azienda Ospedaliera S. Cannizzaro, Catania | Ginecologia ed Ostetricia | Paolo Scollo | Giuseppe Scibilia |
| Casa di Cura La Maddalena, Palermo | Oncologia Medica | Vittorio Gebbia | Valentina Grasso |
| Casa Sollievo della Sofferenza, S.Giovanni Rotondo | Ginecologia ed ostetrica | Giovanni Di Vagno | Francesco Petruzzelli |
| Istituto scientifico oncologico IRCCS, Bari | Oncologia Medica | Vito Lorusso | Agnese Latorre |
| Policlinico Universitario P. Giaccone, Palermo | Oncologia Medica | Nicola Gebbia | Maria Rosaria Valerio |
| Azienda Ospedaliera Mariano Santo, Cosenza | Oncologia Medica | Salvatore Palazzo | Rosalbino Biamonte |
| Azienda Ospedaliera Civile Maria Paternò, Ragusa | Oncologia Medica | Carmelo Iacono | Gianni Occhipinti |
| A.O. Policlinico Mater Domini, Catanzaro | Oncologia Medica | Salvatore Venuta + | Pierfrancesco Tassone |
| P.O.C. Belcolle di Viterbo | Oncologia Medica | Camillo F. Pollera + | Luca Moscetti |
| Ospedale Silvestrini, S.Andrea delle Fratte | Oncologia Medica | Maria Antonietta Colozza | Anna Maria Mosconi |
